# Nurse perceptions of organizational culture and its association with the culture of error reporting: a case of public sector hospitals in Pakistan

**DOI:** 10.1186/s12913-015-1252-y

**Published:** 2016-01-05

**Authors:** Sara Rizvi Jafree, Rubeena Zakar, Muhammad Zakria Zakar, Florian Fischer

**Affiliations:** 1 Institute of Social and Cultural Studies, Sociology Department, University of the Punjab, Lahore, Pakistan; 2 Institute of Social and Cultural Studies, University of the Punjab, New Campus, University of the Punjab, Lahore, Pakistan; 3 Institute of Social and Cultural Studies, Faculty of Behavioral and Social Sciences, New Campus, University of the Punjab, Lahore, Pakistan; 4 Department of Public Health Medicine, School of Public Health, Bielefeld University, P.O. Box 100131, 33501 Bielefeld, Germany

**Keywords:** Organizational culture, Error reporting, Pakistan, Nurse, Public sector

## Abstract

**Background:**

There is an absence of formal error tracking systems in public sector hospitals of Pakistan and also a lack of literature concerning error reporting culture in the health care sector. Nurse practitioners have front-line knowledge and rich exposure about both the organizational culture and error sharing in hospital settings. The aim of this paper was to investigate the association between organizational culture and the culture of error reporting, as perceived by nurses.

**Methods:**

The authors used the “Practice Environment Scale-Nurse Work Index Revised” to measure the six dimensions of organizational culture. Seven questions were used from the “Survey to Solicit Information about the Culture of Reporting” to measure error reporting culture in the region. Overall, 309 nurses participated in the survey, including female nurses from all designations such as supervisors, instructors, ward-heads, staff nurses and student nurses. We used SPSS 17.0 to perform a factor analysis. Furthermore, descriptive statistics, mean scores and multivariable logistic regression were used for the analysis.

**Results:**

Three areas were ranked unfavorably by nurse respondents, including: (i) the error reporting culture, (ii) staffing and resource adequacy, and (iii) nurse foundations for quality of care. Multivariable regression results revealed that all six categories of organizational culture, including: (1) nurse manager ability, leadership and support, (2) nurse participation in hospital affairs, (3) nurse participation in governance, (4) nurse foundations of quality care, (5) nurse-coworkers relations, and (6) nurse staffing and resource adequacy, were positively associated with higher odds of error reporting culture. In addition, it was found that married nurses and nurses on permanent contract were more likely to report errors at the workplace.

**Conclusion:**

Public healthcare services of Pakistan can be improved through the promotion of an error reporting culture, reducing staffing and resource shortages and the development of nursing care plans.

## Background

Although confirmed statistics are missing, evidence from developed countries estimates that billions of dollars are wasted in the health care system annually due to underreporting of errors [1]. In the absence of formal error tracking systems, especially for the developing world, the successful maintenance of a voluntary error reporting culture gains increased importance to ensure patient safety [2]. A favorable error reporting culture is known to be positively associated with a positive organizational culture [3]. The organizational culture in a hospital setting is the product of shared values, attitudes and patterns of behavior which medical practitioners observe during the process of care delivery [4].

Nurse practitioners are more competent in and likely to report errors, compared to other health care providers [5]. Favorable organizational cultures for nurse practitioners have been described as ones with satisfactory coworker communication, higher levels of nurse autonomy, efficient nursing care plans and adequacy in staffing and resources [6, 7]. When nurses and other medical practitioners are facilitated with a positive organizational culture, their commitment to a culture of error reporting and error sharing increases, consequently improving patient safety and reducing mortality rates [8, 9]. WHO also indicates that the organizational culture of a hospital influences health practitioner job satisfaction, role delivery and quality of patient care [10].

More than 95 % of nurses in Pakistan are females [11]. However, nurses are in extreme shortage in the region. The nurse to doctor ratio is at 1:3 and the nurse to patient ratio at 1:50,000 [11]. Nurse problems related to recruitment and retention in the region have been found to be linked to a complex combination of organizational culture issues, including: (i) unsatisfactory coworker relations [12], (ii) the inferior status of the nurse profession and inadequate compensation and benefits [13, 14], (iii) negative nurse identity and high rates of violence against nurses [15, 16], and (iv) the absence of nursing care plans and autonomous work participation [17]. The Health Ministry and the Punjab Healthcare Commission are the official government regulatory bodies that have authority to improve the quality of health care service provision and clinical governance in Pakistan. However, budget allocations and policies for patient safety are neglected areas in the health care organizations of the country [18]. There is no formal error tracking system in public sector hospitals of the region and no formal laws exist to penalize offending practitioners [19]. The curriculum inclusion and monitoring of medical and nursing code of ethics is officiated by the Pakistan Medical and Dental Council (PMDC) and Pakistan Nursing Council (PNC). However, compulsory curriculum inclusion of medical ethics, examination of clinical ethics and formal monitoring of clinical ethics practice is not carried out [20]. Additionally, the status of error reporting from the perspective of public sector nurses in Pakistan has not yet been addressed by research.

### Study objective and relevance of this study

The aim of this paper was to investigate the association between the organizational culture and the culture of error reporting in the public health care sector, as perceived by nurses. It is agreed that policy improvements in the public sector health care services are possible only when there is adequate empirical research about the status quo [21]. Therefore, the findings of this study will attempt to map a plan for improved organizational culture for nurses, and, consequently, facilitate to improve error reporting and patient safety. The study results are expected to be relevant not only for nurses and other medical practitioners working in the public sector, but also for the private health care sector. Our hypotheses for the study were: (1) When organizational culture is favorable, the error reporting culture will be favorable, and (2) When each of the six subscales of organizational culture are favorable (1. nurse manager ability, leadership and support, 2. nurse participation in hospital affairs, 3. nurse participation in governance, 4. nurse foundations of quality care, 5. nurse coworker communication, 6. nurse staff and resource adequacy), the error reporting culture will also be favorable.

## Methods

This study is part of a doctoral dissertation entitled “Nurses’ perceptions of organizational culture and its association with error reporting: A study of tertiary-care public sector hospitals in Lahore”, conducted by the first author of this paper. The study used a descriptive and correlational design, using cross-sectional data and mixed methodology. The qualitative parts of this study have been published earlier [22, 23].

### Setting and sampling procedure

The study was performed in the city of Lahore, which is the capital of the Punjab province in Pakistan. Lahore is the second largest city of Pakistan and the second highest populated city of the country. It is estimated to have more than ten million inhabitants. Data from the website of Pakistan Institute of Medical Sciences official website was used to conveniently sample two geographically spaced tertiary care public sector hospitals from Lahore, out of a total of nine [24]. The two sampled hospitals have been named Hospital A and Hospital B. Both hospitals have high patient turnovers and large in-patient capacities, and are catering to a different set of patients from the rural and urban Lahore District and also from the surrounding villages of Lahore City. Combined, the two hospitals have a large daily out-patient turnover rate of more than 3,800 patients and an in-patient capacity of approximately 1,890 beds.

All registered female nurses who had been working in the hospital for more than one year were sampled. Each designation was sampled, including nurse supervisors, nurse ward heads, nurse instructors, staff nurses and nurse students. Registered nurse students were included in the sample, because they actively perform clinical duties after the first three months of their enrollment as students, and thus they also have rich experience about the state of organizational culture and error reporting in the hospital setting. In Hospital A there is 1 nurse supervisor, 650 staff nurses, 150 nurse ward heads, 20 nurse instructors, and 415 nurse students. In Hospital B (Sheikh Zayed hospital) there is 1 nurse supervisor, 600 staff nurses, 100 nurse ward heads, 13 nurse instructors, and 320 nurse students. Both hospitals combined have a total of 2,270 nurses, with 2 nurse supervisors, 250 nurse ward heads, 33 nurse instructors, 1,250 staff nurses, and 735 nurse students. Yamane’s formulae [25] was used to determine a sample size from an estimated female Punjab nurse workforce of 11,065. Surveys were distributed to the different nurse designations according to their respective weightage. In this way, 35 % of each nurse designation was sampled, including both nurses supervisors, 440 staff nurses, 90 nurse ward heads, 12 nurse instructors, and 260 nurse students.

### Instruments

Survey questions and guidelines were conducted and written in the English language, which is the official academic and working language of the country. Two standardized instruments were used including the “Practice Environment Scale-Nurse Work Index Revised” (PES-NWI) [26], and the “Survey to Solicit Information about the Culture of Reporting” (SSICR) [27]. The validity and reliability of both the PES-NWI [28, 29] and the SSICR [30, 31] has been established by previous research in health care policy improvements. Respondents were provided, through an extensive literature review, with a summarized list of errors that may occur during health care service delivery by medical practitioners (Table 9 in Appendix) [32–34]. A pretest of the questionnaire was conducted with 35 nurse respondents to ascertain any loopholes that could be rectified before the final administration.

Section I of the questionnaire contained 18 questions pertaining to the socio-demographic characteristics of nurses. These questions include age (‘20–29 years’, ‘30–39 years’ and ‘40+ years’), marital status (‘Never married’, ‘Currently married’ and ‘Divorced/separated/widowed’), regional belonging (‘Punjab’, ‘Sindh’, ‘Baluchistan’ and ‘Khyber Pakhtun Khwan’), religion (‘Muslim’, ‘Christian’, ‘Hindu’ and ‘Ahmedi’), total number of children (‘None’, ‘1–2 children’ and ‘3 or more children’), total monthly income (PKR ‘5,000–19,999’, ‘20,000–39,999’ and ‘≥40,000’), place of residence (‘college hostel’, ‘hospital resident colony’ and ‘private home’), highest nursing degree (‘Nursing Diploma’, ‘BSc Nursing’ and ‘MSc Nursing’), current designation (‘Nurse supervisor’, ‘Nurse instructor’, ‘Nurse ward head’, ‘Staff nurse’ and ‘Student nurse’), type of labor contract (‘Permanent’ and ‘Contractual’), employment status (‘Full-time’ and ‘Part-time’), current government grade (‘16 grade’ and ‘17 grade’), additional employment in private sector (‘Yes’ and ‘No’), and additional hours worked at the public sector hospital during the night, day or evening (‘Yes’ and ‘No’).

Section II contained 31 questions from the PES-NWI, which measures the organizational culture of a hospital by a composite score which is aggregated to the unit level [26]. Items on the scale consist of 5 subscales which measure different aspects of organizational culture, including: (1) nurse manager ability, leadership and support (5 items), (2) nurse participation in hospital affairs (7 items), (3) nurse participation in governance (2 items), (4) nurse foundations of quality care (10 items), (5) nurse-coworkers relations (3 items), and (6) nurse staffing and resource adequacy (4 items).

Section III contained seven questions from the SSICR. The questions measure the culture of error reporting in a hospital and indicate how comfortable the respondent is in sharing errors at the workplace with supervisors and coworkers.

Both the scales have a 4 point rating scale, which indicate respondent extent of agreement with each item. The response categories include: 1 = strongly agree, 2 = agree, 3 = disagree, 4 = strongly disagree. The instrument is scored by calculating mean subscale scores and a total composite score for each respondent which can range from 1–4. Higher scores indicate a less favorable organizational culture and culture of error reporting. Two of the seven items from the error reporting variables had to be reverse coded so that the scoring was aligned across all items of the tool. Reliability analysis was conducted for both the PES-NWI and the SSICR to confirm Cronbach’s alphas of scales. A Cronbach’s alpha of above 0.7 is considered a reliable measure for health and social science research [35]. For this study, the overall internal consistency ranged satisfactorily between values of 0.743 to 0.881.

### Data collection

The questionnaire was distributed at the two hospitals in the time period from November 2013 to January 2014. All nurse employees are required to sign an attendance register daily, placed in the offices of their respective nurse ward head, before the start of their shift. Hence all nurse designations visited the nurse ward head office daily at the start of three different shifts of either 08:00 am, 02:00 pm or 08:00 pm. It was deemed suitable to communicate with nurses at this place of contact to recruit interested participants. Nurses were asked to read the cover letter attached to the survey and select a time to complete the survey in a reserved room of the nursing school of each respective hospital. Surveys were filled in nursing school class rooms, specifically reserved for data collection, with 15–30 nurses at a time. The classrooms afforded privacy and a comfortable setting, away from the hospital building, clinical wards, patients and attendants, work pressures, male coworkers and other work-related intrusions. The survey completion time fell between 20–35 min. The first author was present to answer questions related to the survey. Respondents sealed their completed surveys and dropped them in a box before leaving the nursing school. Both the nurse supervisors from each hospital were sampled, 440 surveys were distributed to staff nurses, 90 to nurse ward heads, 12 to nurse instructors and 260 to nurse students (Table 1). In total, 804 questionnaires were distributed, but only 309 nurses pre-booked a time to complete the survey in reserved rooms. All 309 nurses completed the survey and were included in the final analysis (response rate: 34.8 %).

**Table 1:** Nurse samples from Hospital A and Hospital B

Hospital	Nurse supervisors	Staff nurses	Nurse ward heads	Nurse instructors	Students	Total
*Hospital A*						
Actual headcount	1	650	150	20	415	1,236
Target sample	1	220	45	6	130	402
Response	1	126	28	5	42	202
*Hospital B*						
Actual headcount	1	600	100	13	320	1,034
Target sample	1	220	45	6	130	402
Response	1	79	12	6	9	107
*Hospitals combined*						
Total actual headcount	2	1,250	250	33	735	2,270
Total target sample	2	440	90	12	260	804
Total response	2	205	40	11	51	309

### Data analysis

Raw data was first entered into Excel. It was then transferred into SPSS 17.0 for analysis. A significance level of 0.05 was assigned for all statistical analyses. First, a factor analysis was used to reduce data and confirm subscales of relevance for the study. Factor analysis was deemed suitable to validate the NES-PWI for Pakistani public sector hospitals [36–38], as this tool, to the best of researchers’ knowledge, has not been used in the region before. Principal component analysis (PCA) was used with varimax rotation, as guided by previous research [26, 39, 40]. The following conditions for PCA were met: (i) sample size of above 50 cases, (ii) normal distribution, and (iii) all the variables of organizational culture correlating with each other above 0.2 and no correlations of above 0.9 (avoiding fears of multicollinearity).

Descriptive statistics are provided to show socio-demographic and employment characteristics of nurse respondents. Composite scores for subscales of organizational culture and error reporting were calculated [41–45]. Mean scores were also calculated ranging from 1–4 for subscales of organizational culture, composite organizational culture and error reporting. As recommended by literature, scores under the values of 2.5 were considered favorable and scores of above the value of 2.5 were considered unfavorable. Simple bivariate logistic regression and multivariable logistic regression models were used [46–49] to check for the association between: (i) organizational culture and its subscales with error reporting, and (ii) nurse socio-demographic characteristics and error reporting. The aim was to identify the odds of a favorable error reporting culture when organizational culture and its subscales are favorable, and also to identify the odds of a favorable error reporting culture in relation to the socio-demographic features of the nurse. The enter method was used. Variables were recoded into bivariate categories in order to use logistic regression. Organizational culture and its six subscales were recoded with dummy variables of 0 = unfavorable organizational culture and 1 = favorable organizational culture. Error reporting was recoded with a dummy variable of 0 = unfavorable error reporting culture and 1 = favorable error reporting culture. The significance of the main effects was estimated by computing the confidence level for Exp (B) and was presented in form of odds ratios (OR), with accompanying 95 % confidence intervals (95 % CI). Each of the variables of organizational culture, its subscales and the socio-demographic characteristics of nurses were adjusted for nurse age (as a continuous variable), nurse literacy and nurse monthly income.

### Ethical permission, reliability and validity

Ethics committee permission was obtained from the Institutional Review Board, University of the Punjab, and also from the hospitals and nursing institutes where data collection took place. The ethics of the research process for this study were observed diligently, especially in consideration of sampling working women in developing regions, with the absence of structural and legal support [50]. All participants were informed and assured by attaching a cover letter to the questionnaire, describing the objectives of the research and ensuring confidentiality and anonymity. Informed consent was taken from the participants. Private rooms were requested in nursing schools where all surveys were filled and respondents were able to complete the survey in privacy and also to ask any questions related to the survey. Face and content validity of the questionnaire was confirmed through consultation and discussion with senior researchers, nurse supervisors and nurse ward heads. Cronbach’s alpha was used to check the internal consistency and reliability of the items in the instrument [51, 52]. Internal validity was ensured by using a simple random sample so that each participant had an equal chance of selection. Construct validity was assured by using PCA.

## Results

### Factor analysis

The Kaiser-Meyer Olkin test and the Bartlett test were both satisfactory and supportive to conducting a factor analysis. Six factors were extracted by PCA, including all the six subscales for organizational culture represented in the PES-NWI. Results showed 70.8 % of the variance of the construct being studied (i.e. organizational culture). Communalities were extracted (Table 2), and each item explains at least 50 % of the variance of the study construct.

**Table 2:** Factor loadings and communalities from PCA with varimax rotation for organizational culture

Component	Commun-alities
Nurse Participation in Governance	
Q19. Staff nurses are involved in the internal governance of the hospital	.587
Q26. Staff nurses have the opportunity to serve on hospital and nursing department committees	.796
Nurse Manager Ability Leadership and Support	
Q21. An administration who listens to and responds to employee concerns	.738
Q22. A director of nursing highly visible and accessible to staff	.668
Q25. Nursing administrators consult with staff on daily problems and procedures	.710
Q27. A nursing supervisor equal in power and authority to other top level hospital executives	.764
Q38. A head nurse who is a good manager and leader	.642
Q39. A head nurse/supervisor who backs up the nursing staff in decision making, even if the conflict is with a physician	.690
Q40. Supervisors use mistakes as learning opportunities, not criticism	.534
Q41. A supervisory staff that is supportive of the nurses	.753
Q42. Praise and recognition for a job well done	.757
Nurse Participation in Hospital Affairs	
Q20. Many opportunities for advancement of nursing personnel	.768
Q23. Opportunity for staff nurses to participate in policy decisions	.761
Q24. Career development/clinical ladder opportunity	.658
Nurse Foundations for Quality of Care	
Q28. Use of nursing diagnoses	.640
Q29. An active quality assurance program	.726
Q30. An orientation program for newly hired RNs	.737
Q31. Nursing care is based on a nursing, rather than a medical, model	.567
Q32. Patient care assignments that foster continuity of care	.747
Q33. A clear philosophy of nursing that pervades the patient care environment	.700
Q34. Written, up-to-date nursing care plans for all patients	.745
Q35. High standards of nursing care are expected by the administration	.717
Nurse coworker relations	
Q43. A lot of teamwork between nurses and doctors	.651
Q44. Physicians and nurses have good relationships	.738
Q45. Functional collaboration (joint practice) between nurses and physicians	.728
Q46. Enough staff to get the work done	.780
Q26. Staff nurses have the opportunity to serve on hospital and nursing department committees	.796
Nursing Staffing and Resource	
Q47. Enough registered nurses to provide quality patient care	.839
Q48. Adequate support services allow me to spend time with my patients	.712
Q49. Enough time and opportunity to discuss patient care problems with other nurses	.587

### Mean scores of scales

The nurse respondents average composite mean score for organizational culture was ranked favorably at 2.38 (SD = 0.616). The following three areas were ranked unfavorably by nurses (Table 3): (i) error reporting culture (Mean score = 2.62; SD = 0.500), (ii) staffing and resource adequacy (Mean score = 2.56; SD = 0.901), and (iii) nurse foundations for quality of care (Mean score = 2.59; SD = 0.630). The following subscales of organizational culture have been ranked favorably by nurse respondents: (i) nurse participation in hospital affairs (Mean score = 2.34; SD = 0.726), (ii) nurse participation in governance (Mean score = 2.33; SD = 0.781), and (iii) nurse-coworkers relations (Mean score = 2.26; SD = 0.704).

**Table 3:** Mean scores for organizational culture and error reporting scales and organizational culture subscales (score ranges from 1 to 4)

Scale	Mean score	Standard deviation
Error reporting	2.620	.500
Organizational culture	2.384	.616
Nurse participation in governance	2.338	.781
Nurse participation in hospital affairs	2.348	.726
Nurse manager ability, leadership and support	2.296	.632
Nurse foundations for quality care	2.599	.630
Nurse staffing and resource adequacy	2.562	.902
Nurse coworker relations	2.261	.705

### Socio-demographic characteristics of the sample

Of the 309 respondents (Table 4), 202 were from Hospital A (65.4 %) and 107 were from Hospital B (34.6 %). About half of the nurse respondents was in the age group of 20–29 years (*n* = 161, 52.1 %), 87 belonged to the age group of 30–39 years (28.2 %) and 19.7 % (*n* = 61) were 40 years of age and above. The average age was 30.5 years (SD = 9.85). The majority was currently married (*n* = 173, 55.9 %); 130 respondents (42.1 %) were unmarried and 6 respondents (1.9 %) were divorced, widowed or separated. Nearly all respondents belonged to the province of Punjab (*n* = 301, 97.4 %), 6 were from Sindh (1.9 %) and one each (0.3 %) was from Baluchistan and Khyber Pakhtun Khwan, respectively. Most of the nurse respondents were Muslims (*n* = 231, 74.7 %), 76 were Christians (24.6 %) and one respondent each (0.3 %) was Hindu and Ahmedi. More than half of the respondents (*n* = 157, 50.8 %) had no children, 63 had one or two children (20.4 %) and 89 had three children or more (28.8 %). A total of 56 nurses earned between PKR 5,000–19,999 (18.1 %), 154 nurses earned between PKR 20,000–39,999 (49.8 %) and 99 nurses earned more than PKR 40,000 (32.0 %). The average income per month of the nurses in the sample was PKR 33,754. With regard to the place of residence, the majority (*n* = 212, 68.6 %) of nurse respondents lived in private accommodations, 53 (17.2 %) of nurse students lived in college hostel and 44 (14.2 %) lived in the hospital residence colony.

**Table 4:** Socio-demographic characteristics (*n* = 309)

Socio-demographic variables	Unfavorable error reporting n (%)*	Favorable error reporting n (%)*	*n (%)*
Tertiary care public sector hospital			
Hospital A	107 (53.0)	95 (47.0)	202 (65.4)
Hospital B	24 (22.4)	83 (77.6)	107 (34.6)
Age			
20-29 years	28 (17.4)	133 (82.6)	161 (52.1)
30-39 years	68 (78.2)	19 (21.8)	87 (28.2)
40+ years	35 (57.4)	26 (42.6)	61 (19.7)
Marital status			
Never married	26 (20.0)	104 (80.0)	130 (42.1)
Currently married	102 (59.0)	71 (41.0)	173 (55.9)
Divorced/ separated/ widowed	3 (50.0)	3 (50.0)	6 (1.9)
Region			
Punjab	128 (42.5)	173 (57.5)	301 (97.4)
Sindh	2 (33.3)	4 (66.7)	6 (1.9)
Baluchistan	1 (100)	-	1 (0.3)
Khyber Pakhtun Khwan	-	1 (100)	1 (0.3)
Religion			
Muslim	83 (35.9)	148 (64.1)	231 (74.7)
Christian	47 (62.7)	28 (37.3)	76 (24.6)
Hindu	1 (100)	-	1 (0.3)
Ahmedi	-	1 (100)	1 (0.3)
Children			
None	30 (19.1)	127 (80.9)	157 (50.8)
1-2	36 (57.1)	27 (42.9)	63 (20.4)
3+	65 (73.0)	24 (27.0)	89 (28.8)
Income (in PKR)			
5,000-19,999	10 (18.9)	43 (81.1)	56 (18.1)
20,000-39,999	69 (44.8)	85 (55.2)	154 (49.8)
≥40,000	50 (50.5)	49 (49.5)	99 (32.0)
Home residency			
College hostel	16 (30.2)	37 (69.8)	53 (17.2)
Hospital resident colony	18 (40.9)	26 (59.1)	44 (14.2)
Private home	97 (46.6)	111 (53.4)	212 (68.6)

*Frequencies for each subscale add up to the number of participants in the study

A total of 174 (56.3 %) nurses had a highest nursing degree of diploma (Table 5), 120 (38.8 %) had earned a BSc in Nursing and 15 (4.8 %) had an MSc in Nursing. Majority of the nurses (*n* = 205, 66.3 %) had a designation of staff nurse, 51 were student nurses (16.5 %), 40 nurses were ward heads (12.9 %), 11 were nurse instructors (4.2 %) and two were nurse supervisors (0.6 %). A little more than half of the nurse respondents had a permanent position (*n* = 176, 57.0 %). Nearly all the nurse respondents were full-time employees (*n* = 94.8 %). Most of the nurse respondents belonged to the 16 grade government scale (*n* = 256, 82.8 %). 73.8 % (*n* = 228) of the nurses were not working at a private clinic after duty hours at the hospital, whereas 81 (26.2 %) were working at a private clinic. A total of 59 nurses reported having to work additional hours as night duty (19.1 %), 128 reported having to work additional hours in the day (41.4 %) and 124 had to work additional hours in the evening (40.1 %).

**Table 5:** Nurse employment characteristics (*n* = 309)

Employee variables	Unfavorable error reporting n (%)*	Favorable error reporting n (%)*	*n (%)*
Highest degree attained			
Nursing diploma	72 (41.4)	102 (58.6)	174 (56.3)
BSc in Nursing	56 (46.7)	64 (53.3)	120 (38.8)
MSc in Nursing	3 (20.0)	12 (80.0)	15 (4.8)
Current nurse designation			
Supervisor	-	2 (100)	2 (0.6)
Student (+1 year clinical staff)	10 (19.6)	41 (80.4)	51 (16.5)
Staff nurse	93 (45.4)	112 (54.6)	205 (66.3)
Ward head	22 (55.0)	18 (45.0)	40 (12.9)
Nurse instructor	6 (46.2)	7 (53.8)	11 (3.6)
Labor contract			
Permanent	106 (60.2)	70 (39.8)	176 (57.0)
Contractual	25 (18.8)	108 (81.2)	133 (43.0)
Employment status			
Full-time	128 (43.7)	165 (56.3)	293 (94.8)
Part-time	3 (18.8)	13 (81.3)	16 (5.2)
Government grade			
16 grade	106 (42.6)	143 (57.4)	256 (82.8)
17 grade	21 (39.6)	32 (60.4)	53 (17.2)
Private job			
Yes	55 (67.9)	26 (32.1)	81 (26.2)
No	76 (33.3)	152 (66.7)	228 (73.8)
Additional night duty			
Yes	27 (45.8)	32 (54.2 %)	59 (19.1 %)
No	104 (41.6)	146 (58.4 %)	250 (80.9 %)
Additional day duty			
Yes	86 (67.2)	42 (32.8)	128 (41.4)
No	45 (24.9)	136 (75.1)	181 (58.6)
Additional evening duty			
Yes	84 (67.7)	40 (32.3)	124 (40.1)
No	47 (25.4)	138 (74.6)	185 (59.9)

*Frequencies for each subscale add up to the number of participants in the study

### Bivariate analysis

Composite scores for organizational culture, the six subscales of organizational culture and error reporting were calculated (Table 6). The normality assumption was evaluated and scores of all subscales of organizational culture followed the normal distribution. The correlations, using Pearson correlation, between all study variables significantly correlated with values of above 0.3. Correlation coefficients also showed that the variables have a positive relationship and move together in a linear fashion.

**Table 6:** Pearson’s correlation matrix for organizational culture subscales and error reporting

Variables	ER	Governance	NPHA	NMALS	NFQC	NSRA	NCR
ER	1.000						
Governance	.310*	1.000					
NPHA	.406*	.712*	1.000				
NMALS	.324*	.808*	.752*	1.000			
NFQC	.350*	.740*	.743*	.811*	1.000		
NSRA	.630*	.591*	.715*	.676*	.614*	1.000	
NCR	.634*	.472*	.582*	.557*	.509*	.710*	1.000

Notes: *ER* Error reporting, *NPHA* Nurse participation in hospital affairs, *NMALS* Nurse manager ability, leadership and support, *NFQC* Nurse foundations for quality care, *NSRA* Nurse staffing and resource adequacy, *NCR* Nurse coworker relations

*p < 0.01

### Simple bivariate logistic regression

The contingency results for regression have been shown in Table 7. Results for bivariate logistic regression (Table 8) show that the composite organizational culture variable and the subscales of organizational culture all have high odd ratios with favorable culture of error reporting. When organizational culture is favorable, nurses perceived higher odds of error reporting (OR: 2.43, 95 % CI: 1.51–3.92). When nurse participation in governance (OR: 1.83, 95 % CI: 1.16–2.87) and nurse participation in hospital affairs (OR: 2.96, 95 % CI: 1.85–4.70) are favorable, there is a higher odds of error reporting. Similarly, when nurse manager ability, leadership and support (OR: 1.56, 95 % CI: 0.98–2.48), nurse foundations for quality of care (OR: 3.12, 95 % CI: 1.96–4.98), nurse staffing and resource adequacy (OR: 7.83, 95 % CI: 4.64–13.22) and nurse–coworker relations (OR: 6.13, 95 % CI: 3.62–10.37) were favorable, the odds of error reporting was high. The results also show that nurses above the age of 30 years had extremely higher odds of reporting errors (OR: 13.73, 95 % CI: 7.91–23.86). Married nurses (OR: 5.54, 95 % CI: 3.39–9.05) and nurses earning an income of above PKR 40,000 (OR: 2.55, 95 % CI: 1.54–4.21) had higher odds of reporting errors. Also, nurses on a permanent contract (OR: 6.98, 95 % CI: 4.21–11.57) were more likely to report errors.

**Table 7:** Contingency table showing the relationship between organizational culture, its subscales and error reporting (*n* = 309)

Organizational culture and its subscales	Unfavorable error reporting n (%)*	Favorable error reporting n (%)*
Favorable organizational culture	36 (22.9)	121 (77.1)
Unfavorable organizational culture	95 (62.5)	57 (37.5)
Favorable NPG	40 (27.2)	107 (72.8)
Unfavorable NPG	91 (56.2)	71 (43.8)
Favorable NPHA	37 (25.0)	111 (75.0)
Unfavorable NPHA	94 (58.4)	67 (41.6)
Favorable NMALS	58 (30.9)	130 (69.1)
Unfavorable NMALS	73 (60.3)	48 (39.7)
Favorable NFQC	31 (22.6)	106 (77.4)
Unfavorable NFQC	100 (58.1)	72 (41.9)
Favorable NSRA	40 (21.1)	150 (78.9)
Unfavorable NSRA	91 (76.5)	28 (23.5)
Favorable NCR	25 (17.0)	122 (83.0)
Unfavorable NCR	106 (65.4)	56 (34.6)

*Frequencies for each subscale add up to the number of participants in the study

**Table 8:** Simple bivariate logistic regression and multivariable regression for predictors of higher error reporting (*n* = 309)

Variables	OR for higher error reporting (95 % CI)	*p*-value	AOR for higher error reporting (95 % CI)	*p*-value
Organizational culture				
Favorable organizational culture	2.43 (1.51-3.92)	<0.001	3.58 (1.93-6.63)	<0.001
Unfavorable organizational culture	1		1	
Nurse participation in governance				
Favorable NPG	1.83 (1.16-2.87)	0.009	3.33 (1.87-5.95)	<0.001
Unfavorable NPG	1		1	
Nurse participation in hospital affairs				
Favorable NPHA	2.96 (1.85-4.70)	<0.001	5.08 (2.69-9.57)	<0.001
Unfavorable NPHA	1		1	
Nurse manager ability, leadership and support				
Favorable NMALS	1.56 (0.98-2.48)	0.057	2.61 (1.40-4.84)	<0.001
Unfavorable NMALS	1		1	
Nurse foundations for quality care				
Favorable NFQC	3.12 (1.96-4.98)	<0.001	4.83 (2.59-9.02)	<0.001
Unfavorable NFQC	1		1	
Nurse staffing and resource adequacy				
Favorable NSRA	7.83 (4.64-13.22)	<0.001	7.86 (4.18-14.75)	<0.001
Unfavorable NSRA	1		1	
Nurse coworker relations				
Favorable NCR	6.13 (3.62-10.37)	<0.001	5.58 (2.97-10.50)	<0.001
Unfavorable NCR	1		1	
Age				
≥30 years	13.73 (7.91-23.86)	<0.001		
≤29 years	1			
Marital status				
Married	5.54 (3.39-9.05)	<0.001	1.33 (1.17-1.64)	0.001
Not married	1		1	
Income				
≥40,000 PKR	2.55 (1.54-4.21)	<0.001		
≤39,999 PKR	1			
Degree				
BSc in Nursing or above	1.68 (1.07-2.65)	0.025		
Diploma	1			
Designation				
Manager or instructor	2.15 (1.15-4.02)	0.017		
Staff or student nurse	1			
Nature of employment contract				
Permanent	6.98 (4.21-11.57)	<0.001	1.29 (1.14-1.60)	0.001
Contractual	1		1	

Notes: *NPG* Nurse participation in governance, *NPHA* Nurse participation in hospital affairs, *NMALS* Nurse manager ability, leadership and support, *NFQC* Nurse foundations for quality care, *NSRA* Nurse staffing and resource adequacy, *NCR* Nurse coworker relations

### Multivariable logistic regression

Multivariable logistic regression was performed to calculate the adjusted odds ratio (AOR), holding income, education and age (as a continuous variable) as constants (Table 8). Results were highly significant for the odds ratios between error reporting and organizational culture and its subscales. When organizational culture (AOR: 3.58, 95 % CI: 1.93–6.63), nurse participation in governance (AOR: 3.33, 95 % CI: 1.87–5.95), nurse participation in hospital affairs (AOR: 5.08, 95 % CI: 2.69–9.57), nurse manager ability, leadership and support (AOR: 2.61, 95 % CI: 1.40–4.84), nurse foundations of quality of care (AOR: 4.83, 95 % CI: 2.59–9.02), nurse staffing and resource adequacy (AOR: 7.86, 95 % CI: 4.18–14.75) and nurse coworker relations (AOR: 5.58, 95 % CI: 2.97–10.50) were all favorable, the odds of error reporting were significantly higher. Also, married nurses (AOR: 1.33, 95 % CI: 0.17–0.63) and nurses with a permanent contract (AOR: 1.29, 95 % CI: 0.14–0.599) had higher odds of reporting errors.

## Discussion

The results of this study are consistent with expectations that organizational culture and the culture of error reporting are positively associated.

### Mean score results

Our sample respondents ranked the error reporting culture in their hospitals as unfavorable. This has significant implications for other public sector hospitals in the region, since previous research confirms that no formal error tracking systems exist in the Pakistan healthcare setup and also that education in ethics observance and administrative policy measures for promotion of ethical cultures are absent [53]. Currently, there are no all-encompassing state medical laws in the country for the safeguard of either patients or medical and nurse practitioners. There may be several reasons for the absence of an error reporting culture in the hospital settings of Pakistan, as perceived by female nurses in this study. First, senior doctors and physicians have an elevated status and elite labels, which supports bullying and blame-shifting in the hospital setting against junior medical practitioners and nurses [54]. Second, the healthcare sector of the region has a blame culture with punitive action taken against individuals, which prevents individuals from error sharing [55]. Third, nursing is a feminized profession in the region, with female nurses reluctant to report errors due to male dominated and patriarchal work environments and the fear of having to face workplace violence and retribution [19].

Study results also show that hospital staffing and resource adequacy is perceived by nurse respondents as unsatisfactory. Severe shortages in resources and staffing in the public sector hospitals of Pakistan critically undermine efforts of medical practitioners to ensure patient safety [14]. Other research from the region confirms that corruption in the health care sector, with public sector hospitals commonly devoid of basic and life-saving medicines and medical equipment, is responsible for both shortages and high rates of mortality [56]. Of the total national gross domestic product, only 2.9 % is spent on health care and only 1.2 % is allocated to the public sector [57]. Although more than 70 % of the service provision in health care is provided by the private sector in the region, it is estimated that 74 % of the population of Pakistan avail public health care services due to lack of funds [57]. In addition, public sector staffing is lacking due to inadequate budget allocations for hiring and compensation of medical and nurse practitioners. This has led to low enrollment and high rates of immigration [58].

The nurse professional is an integral member of the health care sector who is responsible directly for patient safety, the efficiency of the health care organization and the overall wellbeing of the population [59]. The results of this study, however, highlight that nurse foundations for quality of care in the hospital administration are ranked as unfavorable by respondents. Other research also suggests that Pakistani hospitals are dominated by medical care plans, with little attention to nursing care plans [60]. This may be because nursing is a feminized profession in the region, and male dominated medial administrations give minimal emphasis to nursing care plans for patient care delivery and instead give prominence to medical care plans [61]. Nursing is perceived in patriarchal regions as a care provision, restricted to cleaning, washing and execution of orders passed by doctors and physicians [62]. Non-nurse medical practitioners, medical administrators and patients do not recognize that nurses have medical training and are aware of patient’s medical needs. In this way nursing care plans are not given precedence.

An unexpected finding was that nurses scored other subscales of organizational culture, such as nurse participation in governance, nurse participation in hospital affairs, nurse manger ability, leadership and support, and nurse coworker relations as favorable. Previous studies in the region indicated that nurse autonomy, participation in hospital policy-making and teamwork are extremely unfavorable [12–14, 17]. It may be that nurse perceptions of favorable organizational culture are highly dependent on nurse education, training and exposure. For example, nurses in the sample may not be comparing their work environment to hospitals in developing nations. They could be strongly influenced by cultures in their wider community. Their perception of the organizational culture at their workplace may be better than their domestic and home environments [63]. There is also the disadvantage of attempting to collect survey data about sensitive topics in blame cultures and patriarchal communities, which may influence female respondents in indicating anything negative about governance and management.

### Regression results

This study furthermore confirms, through multivariable regression results, that for an improved error reporting culture, organizational culture and all its six categories (nurse manager ability, leadership and support, nurse participation in hospital affairs, nurse participation in governance, nurse foundations of quality care, nurse-coworkers relations, and nurse staffing and resource adequacy) need to be favorable. Regression results support findings from other international research [43, 64, 65]. Also, our findings show that error reporting is more common amongst nurses who are married and on a permanent contract. Other studies show that married nurses are more likely to participate in studies about errors [66], and this may be because married women are more secure in facing the consequences of error reporting, like blame, shame, retribution and job loss, due to the safety of a dual-income earning household. Furthermore, nurses on permanent contract may be more likely to report errors because of the difficulty in having their state contracts or jobs rescinded, due to extremely slow bureaucratic processing by the government [56].

### Limitations

There are several limitations of this study. The size of the sample is relatively small and excludes public sector tertiary care hospitals across other cities and rural areas of Pakistan. The low response rate of 34.8 % was due to the lack of time available of busy nurses and also the unwillingness of nurses to participate in what was considered a sensitive topic in a male-dominated work organization. In addition, the responses of nurses are guided by their perceptions, which are influenced by their level of education, on-going training and exposure to magnet hospitals. Also, because of the small sample, findings cannot be generalized. Despite the limitations, this study has significant strengths. It is the only research from Pakistan assessing the relationships between nurse perceptions of organizational culture and the culture of error reporting in public sector hospitals. We hope that our study’s findings will have wider macro implications, as improved patient safety is known to help improve overall public health and reduce health costs for the national economy. Additionally, the findings highlight the critical shortages in staffing and resources and the inadequacy of nursing care plans for patient safety culture in the healthcare sector of the region.

## Conclusion and policy recommendations

Findings from our study indicate that a favorable organizational culture, and each of its six components, is important to encourage a favorable culture of error reporting. Our study identifies three main areas that need improvement, including an increase in staffing and resources, developing nursing care plans and improving the error reporting culture. The installation of mandatory and independently monitored error reporting systems, for developing economies like Pakistan, is a process that requires time, fund allocation and structural changes. In such circumstances the voluntary error reporting between coworkers and management and subordinate assumes significance. There is need for independent monitoring of organizational culture and error reporting culture to encourage honest and reliable feedback from healthcare practitioners and nurses.

Nurses, and other health care practitioners must make efforts through union mobilization and gender solidarity, in order to improve (i) their professional status and the development of formal nursing care plans, and (ii) budget allocations for staffing and resource adequacy in the hospital setting [22]. It is also recommended that the nursing profession is propped with overtly manifest networks and facilities in the hospital settings (e.g. separate nursing offices, nurse front-desk enclosures, nurse trays and even nurse assistant ward-boys who define the hierarchy) to emphasize the importance of the nursing care plans [67].

It will be important to invest time and resources in the training of health care employee culture towards a more progressive non-blame culture and encourage a culture of error reporting between coworkers. This may be done through regular and combined training sessions for doctors, physicians and nurses [68]. Apart from the inclusion of error reporting in the code of ethics, and in formal curricula, monitoring and accountability bodies within the public sector healthcare organizations must be established to oversee error sharing and error reporting without individual penalization. It is also recommended that the medical, dental and nursing councils (PMDC and PNC) hold monthly court sessions to protect and defend whistle-blowers who are actively reporting errors and getting penalized for it by coworkers. This will also help to improve error reporting in an immediate manner, until laws are altered.

There is a need for long-term structural improvements that can only be mobilized through the government and top health care administrators, at both the national and provincial level, including the Healthcare Ministry, the PMDC and the PNC and the Punjab Healthcare Commission. It is recommended that: (i) a formal system to track errors is established to monitor and mitigate error making in the public sector of the region, with zero-tolerance for non-reporting and installation of formal error tracking systems, which can be adopted from magnet hospitals in developed countries, and (ii) an increase in budget allocations are made for staffing and resource adequacy. Lastly, medical laws at the state level should be passed with specific attention to (i) penalization of medical and nurse practitioners in the event of ethical violation and (ii) protecting medical and nurse practitioners against wrongful claims by patients.

## Appendix

**Table 9:** Information provided to nurse respondents about errors that may occur in the healthcare setup during service delivery by healthcare providers

Type of error	Example
1. Lack of attentiveness	Nurse did not check wound drains or dressing after surgery
2. Lack of fiduciary concern	Nurse knowledge that doctor is misdiagnosing and failure to question this to prevent patient harm
3. Inappropriate judgment	Lack of skill or knowledge or incorrect application
4. Medication error	Administration of the wrong drug, drug amount or dose of drug to patient
5. Lack of intervention on patients behalf	Failure to provide for patient needs for example advice on mother’s nutritional needs post delivery
6. Lack of prevention	Failure to prevent harm to patient for example in terms of hygiene and infection
7. Mistaken doctor orders	Missing or mistaking an order and as a result causing patient harm
8. Documentation errors	Error in making a chart entry or failure to make a relevant entry all together
